# Multi-species Identification of Polymorphic Peptide Variants via Propagation in Spectral Networks*[Fn FN2]

**DOI:** 10.1074/mcp.O116.060913

**Published:** 2016-09-08

**Authors:** Seungjin Na, Samuel H. Payne, Nuno Bandeira

**Affiliations:** From the ‡Dept. of Computer Science and Engineering, University of California, San Diego, La Jolla, California, 92093;; §Center for Computational Mass Spectrometry, University of California, San Diego, La Jolla, California, 92093;; ¶Pacific Northwest National Laboratory, Richland, Washington 99354,; ‖Skaggs School of Pharmacy and Pharmaceutical Sciences, University of California, San Diego, La Jolla, California, 92093

## Abstract

Peptide and protein identification remains challenging in organisms with poorly annotated or rapidly evolving genomes, as are commonly encountered in environmental or biofuels research. Such limitations render tandem mass spectrometry (MS/MS) database search algorithms ineffective as they lack corresponding sequences required for peptide-spectrum matching. We address this challenge with the spectral networks approach to (1) match spectra of orthologous peptides across multiple related species and then (2) propagate peptide annotations from identified to unidentified spectra. We here present algorithms to assess the statistical significance of spectral alignments (Align-GF), reduce the impurity in spectral networks, and accurately estimate the error rate in propagated identifications. Analyzing three related *Cyanothece* species, a model organism for biohydrogen production, spectral networks identified peptides from highly divergent sequences from networks with dozens of variant peptides, including thousands of peptides in species lacking a sequenced genome. Our analysis further detected the presence of many novel putative peptides even in genomically characterized species, thus suggesting the possibility of gaps in our understanding of their proteomic and genomic expression. A web-based pipeline for spectral networks analysis is available at http://proteomics.ucsd.edu/software.

Microorganisms have evolved their cellular metabolism to generate energy for life in unusual environments (1), and their capabilities are of great interest in the production of renewable bioenergy and could contribute toward managing the world's current energy and climate crisis (2). Genomics studies have increased the number of sequenced bioenergy-related microbial genomes and revealed the possible biological reactions involved in bioenergy production (3). Studies of photosynthetic microorganisms, for example, have yielded insights into how they harvest solar energy and use it to produce bioenergy products (4). Despite this importance of microorganisms, the characterization of diverse microbial phenotypes by proteomics tandem mass spectrometry (MS/MS) has been limited. The dominant approaches for MS/MS analysis heavily rely on the availability of completely annotated genomes (*i.e.* accurate protein databases) (5–7), yet most microorganisms populating the planet have unsequenced or poorly annotated genomes. Thus it remains challenging to identify proteins from environmental and unculturable organisms.

One solution to protein identification in a species with no sequenced genome is to use the genomes of closely related species (8). This requires matching MS/MS data to slightly different peptides in amino acid sequences (polymorphic, orthologous peptides); but matching shifted masses of peptides and their fragment ions is computationally expensive and challenging. Moreover, different species-specific post-translational modifications (PTMs)^1^ can make the cross-species identification more complex. The common computational approach is tolerantly matching *de novo* sequences derived from MS/MS data to the database while allowing for amino acid mutations and modifications (9–11). However, this approach critically depends on good *de novo* interpretations, which are nearly always partially incorrect and yield high-quality subsequences only for a small fraction of all spectra. The blind database search approach, developed to identify peptides with unexpected modifications, can also be used to directly match MS/MS data from unknown species to a database of closely related species, but its utilization is limited because of its exceptionally large search space (12–18). These spectrum-database matching approaches to cross-species identification pose significant challenges in its speed and sensitivity with a huge database, which leads to a much longer search time and more false positive identifications (19, 20).

As a complementary approach to spectrum-database matching, spectral library searching is an emerging and promising approach (21). A spectral library is a large collection of identified MS/MS spectra, and an unknown query spectrum can then be identified by direct spectral matching to the library. The great advantage of this approach is the reduction of search space and the use of fragmentation patterns of peptides. The spectral networks approach expands this concept to the identification of modified peptides in MS/MS data sets (22, 23). Spectral networks do not directly search a database, but groups MS/MS spectra by computing the pairwise similarity between MS/MS spectra of peptide variants and then constructs networks where each spectrum defines a node and each significant spectral pair, highly correlated in the fragmentation pattern, defines an edge (Fig. 1). In spectral networks, identification of spectra belonging to the same subnetwork should be related and thus the peptide sequence for an identified spectrum can be propagated to neighboring unidentified spectra.

We recently reported that a vast number of polymorphic, orthologous peptides across species are present in MS/MS data sets (24). We propose a new approach in cross-species proteomics research that aggregates MS/MS of multiple related species followed by spectral networks analysis of the pooled data to capitalize on pairs of spectra from orthologous peptides, as shown in Fig. 1. This approach does not require advance knowledge of the genomes for all species, and enables the identification of novel, polymorphic peptides across species via interspecies propagation. Compared with previous approaches, cross-species spectral network analysis has two major advantages. First, by matching spectra to spectra instead of spectra to database sequences, spectral networks only consider the sequence variability of peptides present in the samples instead of considering all possible variability across the whole database of related species; thus the performance of spectral networks is independent of database size. Second, the analysis of the set of highly related spectra increases the reliability in identifying polymorphic peptides in that multiple different spectra can support the same novel identification. The utility of spectral networks can be also expanded to the proteomic analysis of microbial communities that often contain hundreds of distinct organisms (25, 26). But despite the success of spectral networks in low complexity data sets (22, 23), the analysis of large multi-species proteomics data requires significantly higher reliability in spectral similarity scores because the number of pairwise spectral comparisons grows quadratically with the number of spectra.

In this work, we present algorithmic and statistical advances to spectral networks to improve its utility with large and diverse spectral data sets. To statistically assess the significance of spectral alignments in pairing millions of spectra, we propose Align-GF (generating function for spectral alignment) to compute rigorous *p* values of a spectral pair based on the complete score histogram of all possible alignments between two spectra. We show that Align-GF successfully addressed the reliability challenge in a large data set analysis and demonstrated its utility by leading to a 4-fold increase in the sensitivity of spectral pairs. Even with this dramatically improved accuracy, a very small number of incorrect pairs in a network can still complicate propagation of annotations. To further progress toward the ideal scenario where each subnetwork consists of only spectra from a single peptide family, we introduce new procedures to split mixed networks from different peptide families and show that these effectively eliminate many false spectral pairs. Finally, we propose the first approach to calculation of false discovery rate (FDR) for spectral networks propagation of identifications from unmodified to progressively more modified peptides. The proposed FDR estimation was conservative and was more rigorous for highly modified peptides, and thus now makes propagation results comparable to other peptide identification approaches.

The cross-species spectral networks techniques proposed here enabled the proteomic analysis of three different *Cyanothece species*, including a strain where the genome sequence is not known. *Cyanobacteria* are one of the most diverse and widely distributed microorganisms and have received significant consideration as satisfying various demands required in bioenergy generation (27). We show that spectral networks can improve peptide identification by up to 38% compared with mainstream approaches, including many polymorphic and modified peptides. Spectral networks could identify peptides with highly divergent sequences (with 7 amino acid mutations) by leveraging networks of variant peptides, and one example subnetwork of species-specific variants of phycobilisome proteins reflects the diversity of photosynthetic light-harvesting strategies (28). Our approach thus demonstrates the potential gains in multi-species proteomics and sets the stage for related developments in higher-complexity metaproteomics samples. Finally, spectral networks revealed many unidentified subnetworks containing only unidentified spectra, thus strongly suggesting the presence of novel peptides that are missing from current protein databases. Although we illustrate the potential of our approach on a specific set of bioenergy-related species, we note that the proposed approach is generic and should be applicable to any other set of related species. The diversity of biologically important protein families could be studied by comparing closely and more remotely related species.

## EXPERIMENTAL PROCEDURES

### 

#### 

##### MS/MS Data Set

MS/MS data from *Cyanothece* sp. ATCC 51142 was previously described (29, 30), and MS/MS data for *Cyanothece* sp. PCC 8801 and *Cyanothece* sp. ATCC 51472 were prepared in a similar manner. Briefly, proteins from *Cyanothece* sp. 51142, *Cyanothece* sp. 8801, and 51472 were treated identically, using 8 m urea and 5 mm tributylphosphine (Sigma-Aldrich, Saint Louis, MO) at 37 °C for 60 min, except for the addition of 1% CHAPS for 45 min prior to digestion in the insoluble protein fraction of 51142. Samples were not alkylated prior to LC-MS/MS analysis. The trypsin-digested samples were separated using strong cation exchange chromatography (SCX) with a PolySulfoethyl A, 200 mm × 2.1 mm, 5 μm, 300-Å column and a 10 mm × 2.1 mm guard column (PolyLC, Inc., Columbia, MD) at a flow rate of 0.2 ml/min. The fractions were subjected to the LC-MS/MS analysis that coupled a constant pressure (5000 psi) reversed phase capillary liquid chromatography system (150 μm i.d. × 360 μm o.d. × 65 cm capillary; Polymicro Technologies Inc., Phoenix, AZ) with a LTQ Orbitrap Mass Spectrometer (Thermo, San Jose, CA). MS1 scans were acquired at 100,000 resolution in the Orbitrap. MS/MS analysis of the ten most abundant precursors was performed at low resolution in the CID ion trap. All RAW files were converted to mzXML using ProteoWizard (ver. 3.0.5655; http://proteowizard.sourceforge.net) which is a set of open-source, cross-platform tools and software libraries that convert various vendor formats to readable standard formats and facilitate proteomics data analysis. All data files and results including annotated mass spectra were deposited on MassIVE (http://massive.ucsd.edu), with the accession MSV000079552.

##### MS-Clustering

Typically, MS/MS data sets contain substantial amounts of redundancy, with multiple spectra coming from the same peptide. We used MS-Cluster (ver. 2.0) (31) to group spectra of the same peptides and compute cluster consensus spectra prior to spectral networks analysis. In brief, MS-Cluster retains peaks in a cluster consensus spectrum based on peak occurrences in the clustered spectra. MS-Cluster reduced the MS/MS data set to a smaller set of spectra, consequently improving the speed of spectral networks by reducing the number of spectra that undergo pairwise comparisons. Originally, the data consisted of 275,756 MS/MS spectra for *Cyanothece* sp. 8801, 481,411 spectra for *Cyanothece* sp. 51142 and 257,442 spectra for *Cyanothece* sp. 51472. MS-Cluster was applied to each species with the precursor window size of 0.1 Da and the fragment ion mass tolerance of 0.4 Da (MS-Cluster does not support ppm tolerance). MS-Cluster yielded 141,140 cluster-consensus spectra for *Cyanothece* sp. 8801, 171,430 cluster-consensus spectra for *Cyanothece* sp. 51142 and 126,148 cluster-consensus spectra for *Cyanothece* sp. 51472. The clustered spectra were used for all subsequent data analysis.

##### Seed Identification and Gold-standard Spectral Pairs

The clustered spectra were searched using MS-GF+ (ver. 9881) (32) to identify seed peptides for propagation in our constructed spectral networks. Spectra were searched with the following parameters: enzyme = trypsin, the number of enzymatic termini = 1/2, the number of missed cleavages = any, precursor mass tolerance = ± 20 ppm, variable modifications = oxidation (Met) and pyro-glu (N-terminal Gln), the number of modifications/peptide = up to 1. The database consisted of 4,335 and 5,239 protein sequences of *Cyanothece* sp. 8801 and 51142, respectively, downloaded from NCBI (August 2014) and was appended with their reversed sequences for target-decoy FDR estimation (33). Finally, the identifications were filtered using a spectrum probability of 1 e-10 (which corresponded to 0.03% spectrum level FDR), resulting in 61,799 peptide-spectrum matches (PSMs). These identifications were also used as the gold standard to evaluate the correctness of spectral pairs: A spectral pair is i) *true* if two peptides are identical, one peptide is a prefix/suffix of the other, or one peptide is a singly modified form of the other, ii) *ambiguous* if two peptides share 12 or more consecutive amino acids or the overlap of theoretical fragment ions of the two peptides is more than 60% iii) *false* for other cases. *Ambiguous* pairs were not counted when evaluating spectral pairs (*i.e.* these were neither *true* nor *false*).

##### Spectral networks analyses

Spectral networks analyses were performed against the clustered spectra from each data set with ± 0.4 Da mass tolerance for fragment ions; mass tolerance for precursor masses was not required as the mass difference between the precursor masses of aligned spectra was assigned to the mass of modification. The maximum possible mass of considered modifications was ± 375 Da. Typically samples digested with trypsin include many partially tryptic peptides (where only one end corresponds to a tryptic cleavage) and peptides containing tryptic cleavages (*i.e.* missed cleavages). Besides amino acid mutations and modifications, spectral networks can detect those truncated/extended peptides from exact tryptic peptides. The mass range of 375 Da would allow the amino acid deletion/extension up to two Trp residues. Initial spectral pairs were accepted if Align-GF *p* value was less than 5 e-9 (see Generation of Align-GF score histogram section), and then were filtered out to restrict the number of precursor masses (100 in our work) contained in a subnetwork (see Splitting mixed subnetworks section below). When seed identifications were loaded into spectral networks for propagation, the edge FDR and Align-GF *p* value threshold could be calculated based on the annotated spectral pairs. Finally, spectral pairs were filtered out to bring the edge FDR to the specified value.

##### Generation of Align-GF Score Histogram

Align-GF computes the score histogram of all possible alignments against a spectrum using the generating function approach, and computes rigorous *p* values of spectral pairs matched to the spectrum based on the score histogram. Each MS/MS spectrum was converted into a Prefix-Residue Mass (PRM) spectrum (scored version of spectrum) using PepNovo (34), where MS/MS peak intensities were converted into log-likelihood scores by considering complementary ions (*b*/*y*), multiply charged ions, neutral losses (-H_2_O and -NH_3_), and 13C isotopes for each peak. In PRM spectra, peaks at masses corresponding to fragmentation of peptide bonds tend to have high scores whereas peaks at other masses tend to have very low scores (or are removed if the resulting likelihood scores are negative), thus improving the signal-to-noise ratio. Align-GF score histograms were computed on converted PRM spectra. A possible alignment against a spectrum *S* is defined as a subset of peaks in *S*, and all possible alignments against *S* can be represented as all possible subsets of peaks in *S* (*i.e.* 2*^N^* possible alignments, where *N* is the number of peaks in *S*). The score of an alignment is calculated as the sum of peak scores in the corresponding subset of *S*, and its probability is calculated as θ*^m^*(1 − θ)^N−^*^m^*, where *m* is the number of peaks in the subset, and θ is the probability of randomly matching a peak in a spectrum, modeled as an independent Bernoulli event (we use θ = 0.05 as estimated by matching randomly generated theoretical peak lists to identified spectra in our data set using fragment mass tolerance of 0.4 Da). Calculated scores and probabilities for all possible subsets of *S* define the Align-GF score histogram as the probability density function for all possible alignments against *S*. Supplemental Fig. S1 shows an example of generating the Align-GF score histogram and illustrates the difference between the score histogram generated by Align-GF and the previously used Gaussian empirical approximation (22, 23).

The calculation of Align-GF histograms can be done rapidly by dynamic programming. Let a variable *D*[*i, t*] be the overall probability of alignments that have score *t* up to the *i*-th peak in *S*. The variable *D*[*i, t*] can be calculated using the following recursion:

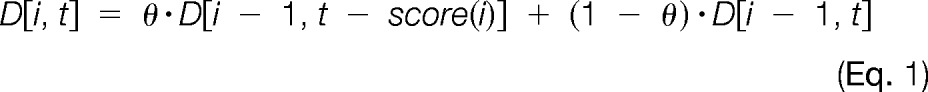
 where θ is the probability of randomly matching a peak and *score*(*i*) is the score of *i*-th peak. The first term in the sum updates the distribution when *i*-th peak is matched, and the second term updates the distribution when *i*-th peak is not matched. *D*[0,0] is initialized to 1 and elsewhere to zero.

Then, if an alignment in *S* has score *T*, the probability that the alignment randomly obtained a score of at least *T* is calculated as follows, where *e* is the last peak index:

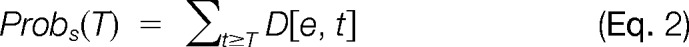


##### Splitting Mixed Subnetworks

In spectral networks, the ideal scenario is that each subnetwork consists of only spectra from a single “peptide family” (where the subnetwork remains connected if using only correct edges), whereas a mixed subnetwork contains spectra from different peptide families. Mixed subnetworks can be caused by incorrect spectral pairs because just one incorrect spectral pair with spectra from distinct peptide families is sufficient to combine the two peptide families into a single subnetwork.

Mixed subnetworks can also be caused by co-fragmented, multiplexed MS/MS spectra. For example, if there is a multiplexed spectrum *S*(*A, B*) including fragment ions from both peptides *A* and *B*, the multiplexed spectrum could possibly be paired with *S*(*A*) and *S*(*B*), and as a result, a subnetwork of peptide *A* would be connected with that of peptide *B* by *S*(*A, B*). Co-fragmentation is commonly observed with 5∼10% of MS/MS spectra often coming from cofragmented precursors (35). Although most multiplexed spectra *S*(*A, B*) have suboptimal Align-GF *p* values against *S*(*A*) or *S*(*A*), frequent cofragmentation could still lead to mixed subnetworks consisting of up to tens of thousands of spectra, which can substantially complicate propagation of annotations.

An intuitive way of splitting mixed subnetworks into single peptide family subnetworks is to remove all incorrect pairs or pairs including multiplexed spectra. But because this information is not available, we limited the potential impact of this issue by restricting the number of unique precursor masses in a single subnetwork to less than a certain predefined value. This was implemented as follows: starting with the best-scoring spectral pair, spectral pairs were introduced into spectral networks by the order of increasing Align-GF *p* values. When two subnetworks would be connected by a newly considered spectral pair, the spectral pair was skipped if the resulting number of unique precursor ion masses in the mixed subnetwork exceeded the predefined value. This process stops when all edges are considered. The rationale is that combining two different subnetworks into a subnetwork larger than a certain size should require highly significant spectral pairs. The *p* values of incorrect pairs are relatively high, and the *p* values of multiplexed spectra would be also high because when the multiplexed spectrum is aligned to spectra of its isolated component peptides, fragment ions from the other peptide would remain unmatched. This approach significantly improved the quality of the spectral network and supplemental Fig. S2 shows the gains in performance.

##### FDR from Propagation

The errors in both seed identification and spectral pairs are involved in the overall error of propagation of identifications in spectral networks. A propagated identification would be correct only if i) the seed identification is correct and ii) all propagation steps are correct. Thus, propagation FDR can be estimated as the probability that, given a seed and all propagation steps, at least one of these is incorrect. Supplemental Fig. S3 illustrates the propagation FDR calculation. FDR is calculated for every propagation step and *FDR_n_* after the *n*-th propagation to the current node is calculated as follows:

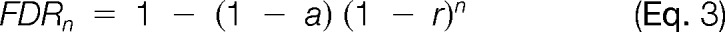
 where *a* is the FDR in seed identification, and *r* is the FDR in spectral pairs. Note that *a* is known because seeds are given (we used *a* = 0.0003), and *r* can be estimated using spectral pairs annotated by the seeds (we used *r* = 0.005). This formula implements the intuition that identifications via more steps of propagations would include a greater error because of accumulated edge errors.

The aggregate FDR for overall identification up to current step was calculated as follows

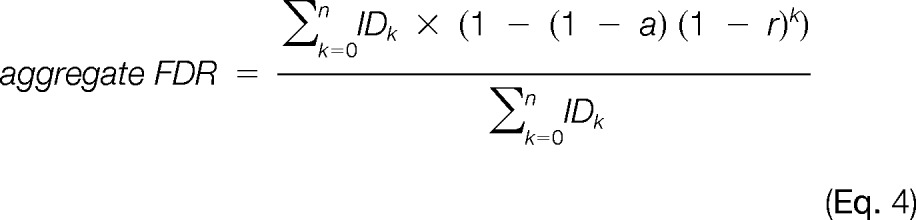
 where *ID_k_* is the number of identifications added at *k*-th propagation

The propagation procedure is stopped when the aggregate FDR reaches a prespecified FDR threshold.

##### Modification Localization

Once a peptide sequence from an identified spectrum is propagated to an unidentified spectrum, new identification to the unidentified spectrum can be determined. A putative modification mass is calculated as a precursor mass difference between the two spectra, and then the position of the modification in the propagated peptide is assigned as follows. Every amino acid in the peptide is assumed as a possible modification site. A modification to each amino acid would generate a theoretical spectrum, where the masses of fragment ions are shifted according to the modification mass and location. All theoretical spectra are scored against the unidentified spectrum, and then the modification site with the highest score is determined. This new identification would be also propagated to other unidentified spectra. Supplemental Fig. S4 illustrates the procedure.

##### MS-GF+ and MODa searches

For the performance comparison with established tools, two searches were conducted. One was a standard database search, MS-GF+ (ver. 9881) (32), and the other was an unrestrictive blind modification search, MODa (ver. 1.23) (18). MS-GF+ search parameters were as follows: enzyme = trypsin, the number of enzymatic termini = 1/2, the number of missed cleavages = any, precursor mass tolerance = ± 20 ppm, C13 errors in precursor mass = up to 2, variable modifications = oxidation (Met) and pyro-glu (N-terminal Gln), the number of modifications/peptide = up to 2. MODa search parameters were as follows: enzyme = trypsin, the number of enzymatic termini = 1/2, the number of missed cleavages = any, precursor mass tolerance = auto correction, fragment mass tolerance = ± 0.4 Da, modification mass size = ±200, the number of modifications/peptide = any. The database consisted of 4,335 and 5,239 protein sequences of *Cyanothece* sp. 8801 and 51142 downloaded from NCBI (August 2014) and was appended with their reversed sequences. Finally, peptide identifications were obtained at FDR 1% using target-decoy FDR estimation (33).

##### Software Implementation

Algorithms and software for spectral networks analysis were integrated to a pipeline (supplemental Fig. S5), which is available at http://proteomics.ucsd.edu/software. The spectral networks pipeline supports various MS/MS spectral types: (1) mzML, mzXML, mgf, and pkl as file formats; (2) CID, HCD, and ETD as fragmentation methods; (3) high-resolution MS/MS instruments such as Q-Tof and Q-Exactive. The results page provides a tabular list view for peptide/protein report, which can be filtered and arranged by various criteria, and a graphical view for the annotation of MS/MS spectra.

## RESULTS

**Fig. 1.**
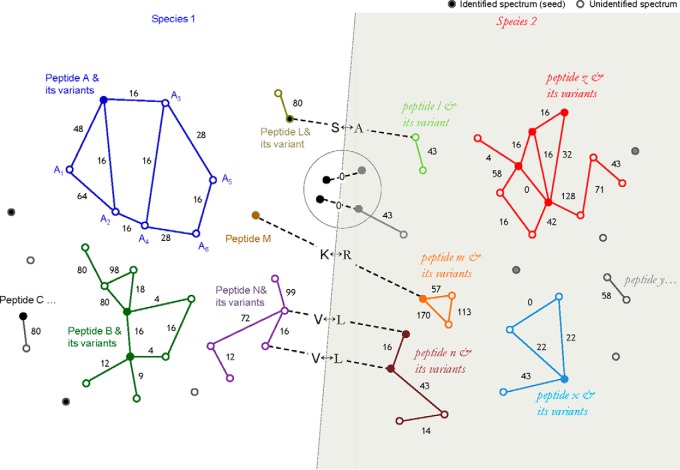
**Overview of multi-species spectral networks.** Nodes represent individual spectra and edges between nodes represent significant pairwise alignment between spectra; edges are labeled with amino acid mutations (dotted edges) or parent mass differences (solid edges). In spectral networks, a peptide and its related variants are ideally grouped into a single subnetwork. If at least one spectrum in a subnetwork is annotated (filled node), all the neighboring spectra (unfilled nodes) can potentially become identified by propagating the annotation over network edges. For example, all spectra in the subnetwork of “peptide A” (top left, blue network) can be annotated via up to three iterative propagations, first from A to {A_1_, A_2_, A_3_}, second from {A_2_, A_3_} to {A_4_, A_5_}, and third from {A_4_, A_5_} to A_6_. This paradigm can be equally applied to cross-species data analysis, as “peptide L” identified in species 1 (top middle, olive-colored network) is propagated to a node unidentified in *species* 2, identifying its orthologous “*peptide l*”, with a serine to alanine polymorphism. Thus, spectral networks enable the detection of orthologous peptide pairs between different species.

Biohydrogen is an emerging renewable and green energy source, and several microalgae and bacterial species are studied as model organisms for photobiological hydrogen production. *Cyanothece* sp. 51142, a unicellular, diazotrophic cyanobacterium, is capable of performing oxygenic photosynthesis during the day and nitrogen fixation at night, resulting in remarkably high rates of hydrogen production under aerobic condition (36). Studying related *Cyanothece* species is of great interest to identify variants with potentially improved biofuel production mechanisms. However, as is generally the case with organisms with poorly annotated genomes, proteomics analysis for various environmental isolates is challenging because of the lack of accurate and complete proteomes. Here we show how this proteomics challenge can be addressed for *Cyanothece* 51472 (which lacks a sequenced genome) by using spectral networks of MS/MS data of related species (*Cyanothece* 8801 and *Cyanothece* 51142) (see Fig. 1).

**Fig. 2.**
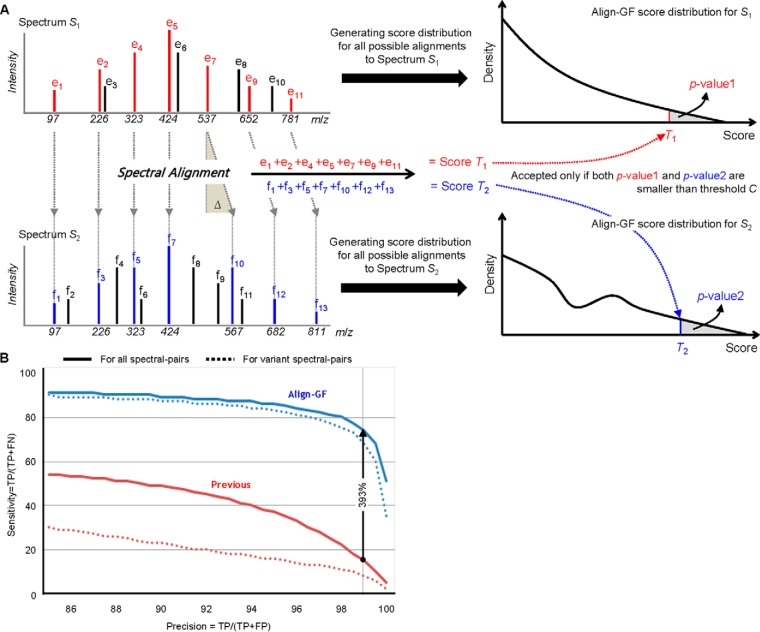
**Align-GF algorithm overview.** *A*, Matching a spectral pair. Two MS/MS spectra are aligned allowing up to one modification, matching red peaks in *S*_1_ with blue peaks in *S*_2_. The total score *T*_1_ of red peaks indicates how well *S*_1_ is explained by this alignment, and the same for blue peaks in *S*_2_ and *T*_2_. Align-GF assesses each score, *T_i_*, based on the histogram of all possible alignments against the corresponding spectrum, *S_i_*, and computes its *p* value - the probability of randomly matching a spectrum with a score at least as high as *T* (*e.g.* the probability that *T*_1_ is the result of a random match to *S*_1_). More details are provided under Experimental Procedures. If both *p* values of *T*_1_ and *T*_2_ are less than the predefined cutoff, the two spectra are accepted as a spectral pair. Edges in spectral networks are thus defined by spectral alignments between spectra *S_i_* and *S_j_. B*, Align-GF performance. The correctness of spectral pairs was evaluated using MS-GF+ annotations. The blue curve represents the performance of Align-GF, whereas the red curve represents the previous method. The previous method assumed that alignment scores between unrelated spectra conform to a Gaussian distribution (22, 23). The dotted curve shows the performance in detecting variant pairs, in which one peptide is a prefix/suffix or a singly modified/mutated form of the other.

Analogous to the pairwise alignment of sequence reads in genome assembly, the fundamental operation in spectral networks is pairwise alignment of spectra to spectra. Starting with over 1 million spectra from all three *Cyanothece* species, the search space contains ∼500 billion spectrum/spectrum pairs where >99.9% are expected to be incorrect. To address this challenge we first used MS-Cluster (31) to condense redundant spectra into 438,718 representative consensus spectra and then scored pairwise alignments between consensus spectra using our new Align-GF approach to compute rigorous *p* values of spectral alignments (see Fig. 2*A* and supplemental Fig. S1; details under Experimental Procedures). Align-GF successfully detected a very high fraction of all true spectral pairs from many billions of false positive pairs, resulting in a 393% increase in sensitivity at 99% precision in comparison with the previous method (see Fig. 2*B* and supplemental Fig. S1*D*). In particular it should be noted that Align-GF detected pairs of variant peptides (*e.g.* mutated, modified or truncated) equally well as pairs of identical peptides (dotted curve in Fig. 2*B*). Generally, pairing the spectra of slightly different peptides is more valuable because they capture post-translational modifications and sequence divergence between species, but it is more difficult than pairing spectra of identical peptides.

As pairs are aggregated into networks, ideal subnetworks should consist of only spectra from related variants of one peptide, a kind of “peptide family.” However, incorrect spectral pairs connect otherwise correctly disjoint subnetworks. Even at Align-GF's 99.99% specificity and 99% precision, the remaining high-scoring incorrect spectral pairs can create large mixed networks. Two issues generally lead to mixed subnetworks: false positive spectral pairs and co-fragmented peptides in MS/MS spectra (37). We addressed this issue using a network-splitting procedure to separate mixed networks by limiting the number of distinct precursor masses per subnetwork (Experimental Procedures and supplemental Fig. S2). Network splitting removed 37% of incorrect spectral pairs from the spectral network and resulted in ∼5000 more properly disjoint subnetworks while removing only 0.2% of correct pairs. In contrast, a corresponding adjustment of the Align-GF threshold eliminates only ∼2% of incorrect pairs (∼18× less) with the same sensitivity loss.

The final spectral network covered 242,341 spectra from the three *Cyanothece* species (55% of the input consensus spectra) combined into 33,188 subnetworks with 99.5% precision of spectral pairs. Spectral networks yield additional peptide identifications via propagation across spectral pairs and retain final identifications at FDR 1% (see Experimental Procedures for details). Prior to propagation 16,932 annotated subnetworks (covering 180,236 spectra) contained at least one identified spectrum (out of 61,799 PSMs used as seed annotations). The remaining 16,256 unidentified subnetworks (62,105 spectra) did not contain any identified spectra.

### 

#### 

##### Spectral Networks Across Species

**Fig. 3.**
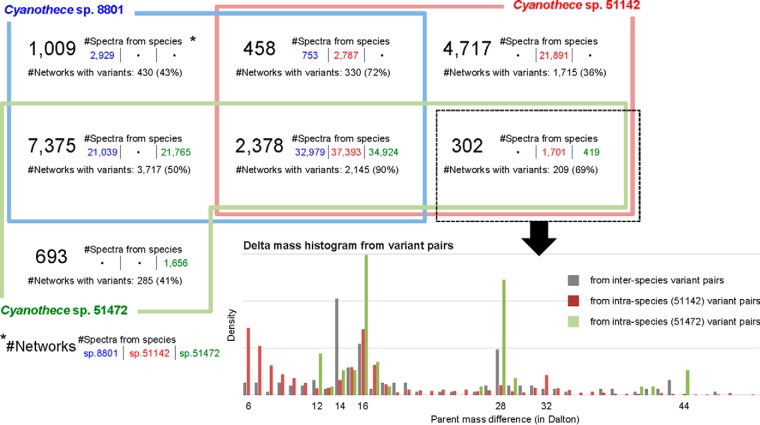
**Annotated spectral networks.** This Venn diagram classifies 16,932 annotated subnetworks into seven groups by combinations of species based upon the origin of the spectra in each subnetwork; blue represents *Cyanothece* sp. 8801, red *Cyanothece* sp. 51142, and green *Cyanothece* sp. 51472. There are 2,378 subnetworks that include spectra from all species; 97% of all networked spectra from *Cyanothece* 51472 (with unknown genome) were contained in multi-species subnetworks. For each group, two properties are shown: 1) #*Spectra from specie*s is the number of spectra in the group from species 8801 at the first column, 51142 at the second, and 51472 at the third, respectively; 2) #*Networks with variants* is the number of subnetworks that include variant pairs - spectral pairs in which the parent mass difference between the two spectra is larger than 5 Da. In the right bottom, the delta mass histogram is shown for variant pairs in the group that includes spectra from species 51142 and 51472 (dotted rectangle), where the gray distribution was calculated from interspecies pairs.

To evaluate the effectiveness of spectral networks across species, we separated the 16,932 annotated subnetworks into 7 groups (see Fig. 3). This readily revealed that species 8801 and 51472 were closely related in that subnetworks across the two species were most frequently found (covering >95% of all networked spectra from species 8801 and 51472), whereas species 51142 was comparatively distant from the other species in that intraspecies subnetworks were more frequently observed (33% of all networked spectra from species 51142).

We next focused on subnetworks containing variant pairs - spectral pairs where the parent mass difference between the spectra in the pair is larger than 5 Da (to avoid ambiguities because of precursor mass errors). It should be noted that subnetworks consisting of spectra from multi-species are more likely to contain peptide variants because orthologous peptides are commonly observed in proteomics data from different species. For example, ∼90% of the 2,378 subnetworks consisting of spectra from all three species contained variant pairs, compared with 36% for those including only spectra from species 51142. In Fig. 3, the presence of orthologous peptides is also supported by a strong peak at 14 Da (corresponding to 8 different amino acid mutations: Asp/Glu, Gly/Ala, Ser/Thr, Asn/Gln, Asn/Lys, Thr/Asp, Val/Leu, and Val/Ile) in the delta mass histogram from interspecies pairs. Moreover, subnetworks across all three species contain a majority of spectra and the spectral count from each species is evenly abundant, indicating that abundantly observed peptides in one species have their orthologous ones that are also abundantly observed in different species.

**Fig. 4.**
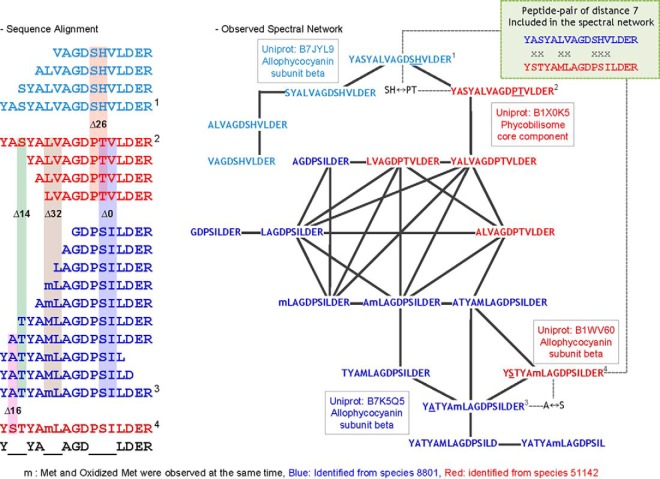
**Multi-species spectral network with species-specific mutations.** Twenty-three different peptides from four versions of “allophycocyanin subunit beta” protein constructed a single subnetwork. Pairwise spectral alignments that pass the Align-GF cutoff are shown as edges connecting the peptide sequences. The blue series of peptides were from species 8801 (Uniprot: B7K5Q5_CYAP8 in dark blue and B7JYL9_CYAP8 in light blue), and red series from 51142 (Uniprot: B1WV60_CYAA5 in dark red and, B1X0K5_CYAA5 in light red). Through this dense network, we are able to connect highly divergent peptides, *e.g.* peptides 1 and 4 differ by seven amino acids.

One example of a multi-species spectral network with species-specific mutations is shown in Fig. 4. Twenty-three polymorphic peptides from allophycocyanin proteins, two from species 8801 (blue series) and two from species 51142 (red series), constructed a single subnetwork. Paired peptides differed by one and two amino acid mutations, oxidation or truncation events. These chromophore containing phycobilisome proteins are key components of light harvesting complexes, found within specialized thylakoid membranes, and which facilitate the capture and transfer of light energy into the cellular reaction centers. Polymorphisms in these proteins have significant implications on energy harvesting and transfer, photosynthetic efficiency, and hence production of downstream bioenergy related molecules. As such, they are of high interest for optimizing synthetic bioenergy production. This subnetwork is highlighted to show that two peptides with seven amino acid mutations were connected via intermediate mutated and truncated peptides, showing how spectral networks can detect distantly related peptide variants.

##### Peptide Identification Via Propagation

Propagating annotations is a unique feature in spectral networks, and can lead to significant increases in the number of identified spectra, including multiply modified peptides. The accuracy of propagation relies on both the accuracy of seed identifications and the reliability of spectral pairs. For example, if seed identifications were obtained at FDR 1%, all identifications propagated from the 1% false-positives must be also incorrect. Additionally, because spectral networks contain a small fraction of incorrect edges, errors also accumulate with more steps of propagation. After a systematic analysis of the errors in propagation, we propose a new approach to estimate the FDR of peptide identifications in spectral networks so that we can control the propagation procedure and make the identification results comparable to other search tools (Experimental Procedures and supplemental Fig. S3).

**Fig. 5.**
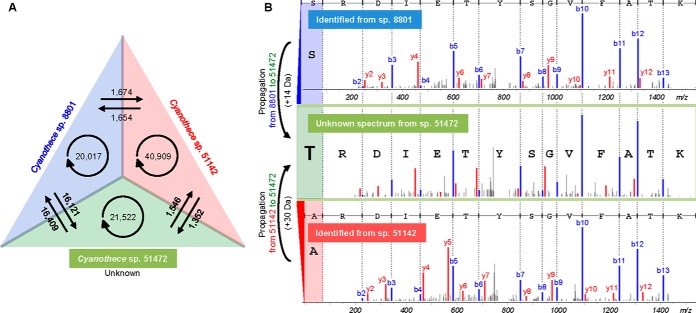
**Propagation over multi-species spectral networks.** *A*, The numbers of intraspecies (circled arrows) and interspecies (arrows between colored triangles) propagations are shown. *B*, Propagation into unknown species 51472 identified novel, orthologous peptides. The fragment ion alignment is shown between a spectrum of a novel peptide (middle) identified by propagation from two seed peptides from different species in the same subnetwork. The two seeds were identified from dihydrolipoyl dehydrogenase in each species.

In the spectral networks of three *Cyanothece*, we started propagation with 61,799 seed identifications from MS-GF+ (32). Nonannotated nodes received their annotation from the incoming edge with the lowest Align-GF *p* value (supplemental Fig. S6). Spectral networks identified 121,204 additional spectra at FDR 1%, leading to the identification of 95% of spectra in annotated subnetworks. Fig. 5*A* shows the numbers and directions of propagations across species. The intraspecies propagations (shown as the circled arrows) mainly identify modified peptides, whereas interspecies propagations identify orthologous peptides. We note that propagation through spectral networks increases the confidence and sensitivity in the assignment of modified/mutated peptides, as spectral pairs provide additional evidence that the corresponding spectra contained highly correlated peptide fragmentation patterns. This strength is maximized in multi-species analysis, where grouping multiple variants of a peptide across species increases the chance of reliably identifying novel peptides through alignment with multiple related peptides. As illustrated in Fig. 5*B*, the novel peptide identification in sp. 51472 is supported by high correlation with both seed identifications from sp. 8801 and 51142.

Multistep propagation allows spectral networks to be extremely flexible in the identification of highly modified peptides and supplemental Fig. S7 shows an example of identifying a triply modified peptide. Propagation via the network avoids the large search space of blind searches (which allows one modification of any mass anywhere in the database) by allowing for any number of modifications but only as derived from seed sequences. This is a great advantage in that the performance of blind database searches tends to degrade rapidly with increasing numbers of modifications and sites per peptide, in both the accuracy of peptide identifications and the speed of the searches (19, 20).

##### Competence in Identifications

**Fig. 6.**
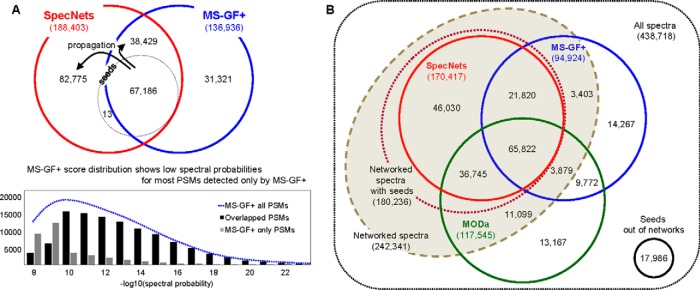
**Identification performance.** All of the identifications were obtained at FDR 1%. *A*, Comparison with MS-GF+. MS-GF+ score distributions show the difference between PSMs that spectral networks also identified (black) and PSMs that spectral networks lost (gray). *B*, Identification on networked spectra. Nonnetworked spectra in seeds of spectral networks were excluded from the comparison.

To contrast the identification performance of spectral networks with mainstream approaches, the results were compared with the standard database search tool MS-GF+ (32), and the blind search tool MODa (18) (see Experimental Procedures). Spectral networks initially used the subset of MS-GF+ PSMs as seeds for propagation (at FDR 0.03%) and increased the number of identifications in networked spectra by ∼180% at FDR 1%. Consequently, spectral networks resulted in a total of 38% more PSMs than MS-GF+ (Fig. 6*A*). Further examination of the 31,321 PSMs identified only by MS-GF+ revealed that most PSMs had low spectral probabilities (in Fig. 6*A*), and are thus most likely derived from less informative spectra.

Spectral networks take advantage of spectral pairs and emphasize the improvement of identification performance on networked spectra. In Fig. 6*B*, spectral networks show the significant improvement on networked spectra, resulting in 80 and 45% more PSMs than MS-GF+ and MODa, respectively. The loss of sensitivity in spectral networks mainly occurred at nonnetworked spectra, which have poor PSM scores (Fig. 6*A*). This identification inconsistency for poor quality spectra between search tools is common in MS/MS data analyses (38).

## DISCUSSION

Microorganisms are very diverse and their proteomics analyses have been limited because of the lack of sequenced genomes. Multi-species spectral networks provide a new way to identify microbial peptides and proteins. The spectral networks analysis of three related *Cyanothece* confirmed that orthologous peptides were commonly observed in multi-species MS/MS data sets, and identified many polymorphic peptides across species, leading to more identifications than other approaches, even for organisms without a sequenced genome. The algorithmic and statistical advances in spectral networks successfully addressed reliability challenges in large-scale analysis of multiple species data sets. Align-GF rigorously assessed the statistical significance of each spectral alignment using the score distribution of all possible alignments, and thus provided excellent separation between correct and incorrect spectral pairs. Align-GF detected spectral pairs of variant peptides equally well as those of identical peptides, resulting in a 4-fold increase in sensitivity. This core improvement significantly expands the utility of spectral networks. We also, for the first time, proposed the FDR estimation of propagating annotations through a spectral network. Although some approaches have proposed the identification of modified peptides by propagating annotations (39–41), none has fully addressed FDR estimation. Our approach ensures a conservative estimate of the FDR and is increasingly rigorous for higher-modified peptides. Most importantly, the FDR estimation also makes spectral networks results comparable with other methods and reveals that the resulting spectrum identification rates can be nearly complete (currently at 95%) as soon as there is at least one seed in a subnetwork.

Spectral networks emphasize the importance of analyzing spectra as a group, which significantly increases both the number of identified spectra and their confidence. Nevertheless, we acknowledge that spectra that do not group into spectral networks or subnetworks which lack a seed annotation are currently not suitable for spectral networks analysis. Missing seeds may be because of database or search tool limitations, and using several tools (including spectral networks *de novo* sequencing approaches (42)) or expanded databases may help address the problem. However, in our multi-species analysis, we note that a significant portion of unidentified subnetworks consisted of spectra from multi-species and contained variant pairs (supplemental Fig. S8), strongly suggesting that these unidentified subnetworks contain novel peptides that are still missing from the database. The properties of unidentified subnetworks are similar to those of annotated subnetworks.

Recent developments in high mass accuracy tandem mass spectrometry (*e.g.* HCD) have significantly improved spectrum identification performance by reducing the chance of randomly matching fragment ion peaks (43). Our approach also supports the analysis of various types of high mass accuracy MS/MS data (see Software Implementation under Experimental Procedures), and users can specify low tolerances for fragment ion masses according to the instrument accuracy. Although the data analyzed here was not obtained using high mass accuracy MS/MS, previous spectral networks approaches have been applied to such types of data and similar gains (44) are expected when applying the current approach to high mass accuracy MS/MS.

Although current approach was based on the similarity in fragmentation pattern between MS/MS spectra within the specified mass difference, the utilization of additional information could further improve the performance of spectral alignment. For example, although we allowed any mass of modifications to search for all known and even possibly unknown types of modifications at once, more targeted analysis could be performed by allowing only user-defined modifications or all modification types known in Unimod database (http://www.unimod.org). This could filter out many incorrect spectral alignments whereas possibly increasing the confidence of modified peptide identifications. Retention time differences (39, 45) could also be used to potentially reduce incorrect spectral alignments. Finally, the impact of multiplexed spectra causing incorrect grouping of spectra into mixed networks could also be potentially improved by investing and thresholding precursor ion fraction (37), defined as the fraction of the targeted precursor ion in the MS/MS isolation window.

### 

#### 

##### Competing Financial Interests

N.B. has an equity interest in Digital Proteomics, LLC, a company that may potentially benefit from the research results; Digital Proteomics LLC was not involved in any aspects of this research. The terms of this arrangement have been reviewed and approved by the University of California, San Diego in accordance with its conflict of interest policies.

## Supplementary Material

Supplemental Data

## Footnotes

^1^ The abbreviations used are:

PTM: post-translational modification
PSM: peptide-spectrum match
FDR: false discovery rate
PRM: prefix-residue mass
CID: collision induced dissociation
HCD: higher-energy C-trap dissociation
ETD: electron transfer dissociation.
