# Supplementary material for: Multi-species Identification of Polymorphic Peptide Variants via Propagation in Spectral Networks
Source: Mol Cell Proteomics. 2016 Sep 8;15(11):3501–12. doi: 10.1074/mcp.O116.060913 (PMC5098046; doi:10.1074/mcp.O116.060913)
Supplement: Supplemental Data [file supp_15_11_3501__index.html]

Multi-species identification of polymorphic peptide variants via propagation in spectral networks — Multi-species Identification of Polymorphic Peptide Variants via Propagation in Spectral Networks — Cross-species Spectral Networks — Supplemental Data 

# Multi-species Identification of Polymorphic Peptide Variants via Propagation in Spectral Networks

## Supplemental Data

- Supplemental Figures (.pdf, 805 KB) - Supplemental Figures
- Supplemental Data (.xlsx, 57.3 MB) - Supplemental Data
