# Supplementary material for: Multi-species Identification of Polymorphic Peptide Variants via Propagation in Spectral Networks
Source: Mol Cell Proteomics. 2016 Sep 8;15(11):3501–12. doi: 10.1074/mcp.O116.060913 (PMC5098046; doi:10.1074/mcp.O116.060913)
Supplement: Supplemental Data [file 10.1074_O116.060913_mcp.O116.060913-1.pdf]

Supplemental Figures

Figure S1.

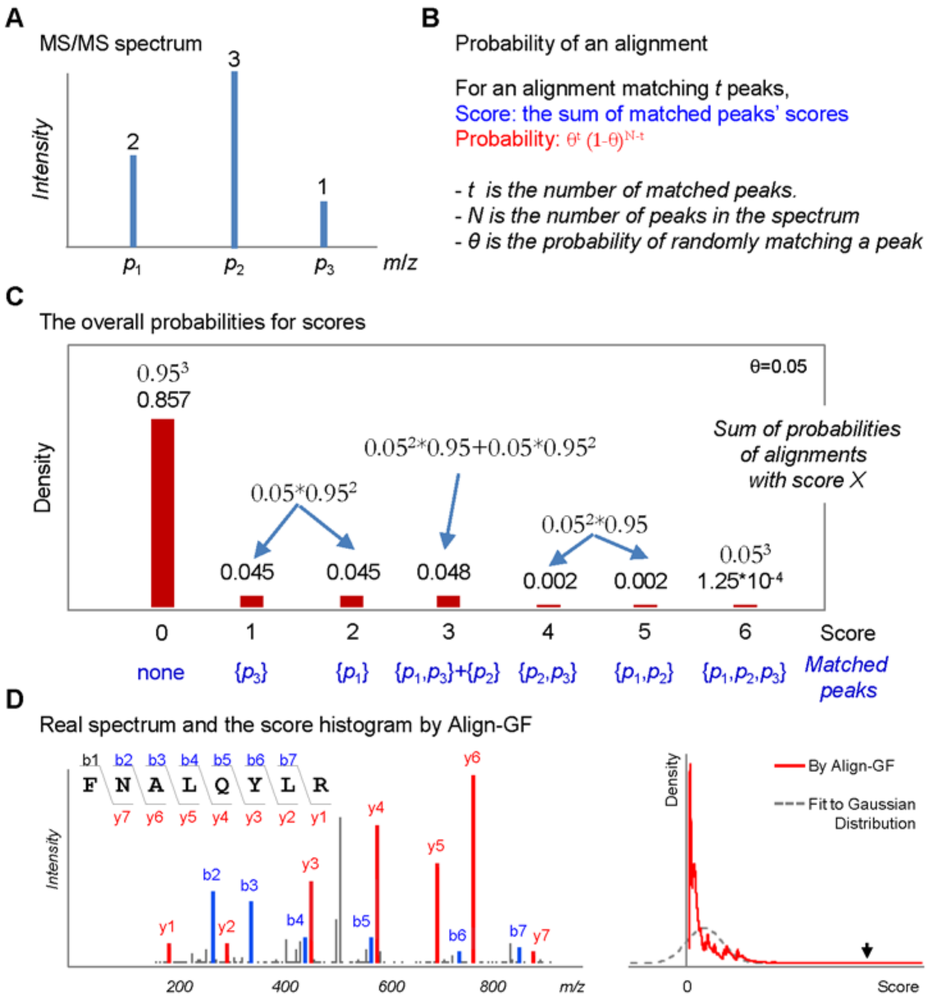

Generating Align-GF score histogram. Given a spectrum consisting of three peaks, $\{p_1, p_2, p_3\}$, whose scores are $\{2,3,1\}$, respectively, there are 8 different possible alignments to the spectrum (all subsets for peaks in the spectrum). A score for each alignment is calculated as the sum of peak scores and its probability is also calculated, where matching a peak in a spectrum is considered as an independent Bernoulli event with probability θ (θ=0.05 in our work). For example, there are two alignments with score 3, $A_1 = \{p_1, p_3\}$ and $A_2 = \{p_2\}$. A_1 has two matching peaks and one unmatched peak and its probability is calculated as $\theta^2(1-\theta)$, while A_2 has one matching peak and its probability is $\theta(1-\theta)^2$. The overall probability for alignments with score 3 is calculated as the sum of the two, $\theta^2(1-\theta)+\theta(1-\theta)^2$. (C) Calculated scores and probabilities for all alignments define the score histogram as a probability density function. (D) A real spectrum (left) and its Align-GF score histogram (right, red) are shown. Previous spectral networks algorithms estimated the significance of spectral pairs based on empirical approximation of a score distribution of observed alignments, where it was assumed that the score between a spectrum and any unrelated spectrum in the dataset conforms to a Gaussian distribution. In the right panel, the gray curve represents the approximation to Gaussian distribution, which was not a proper fit to the data and thus did not accurately assess the significance.

Figure S2.

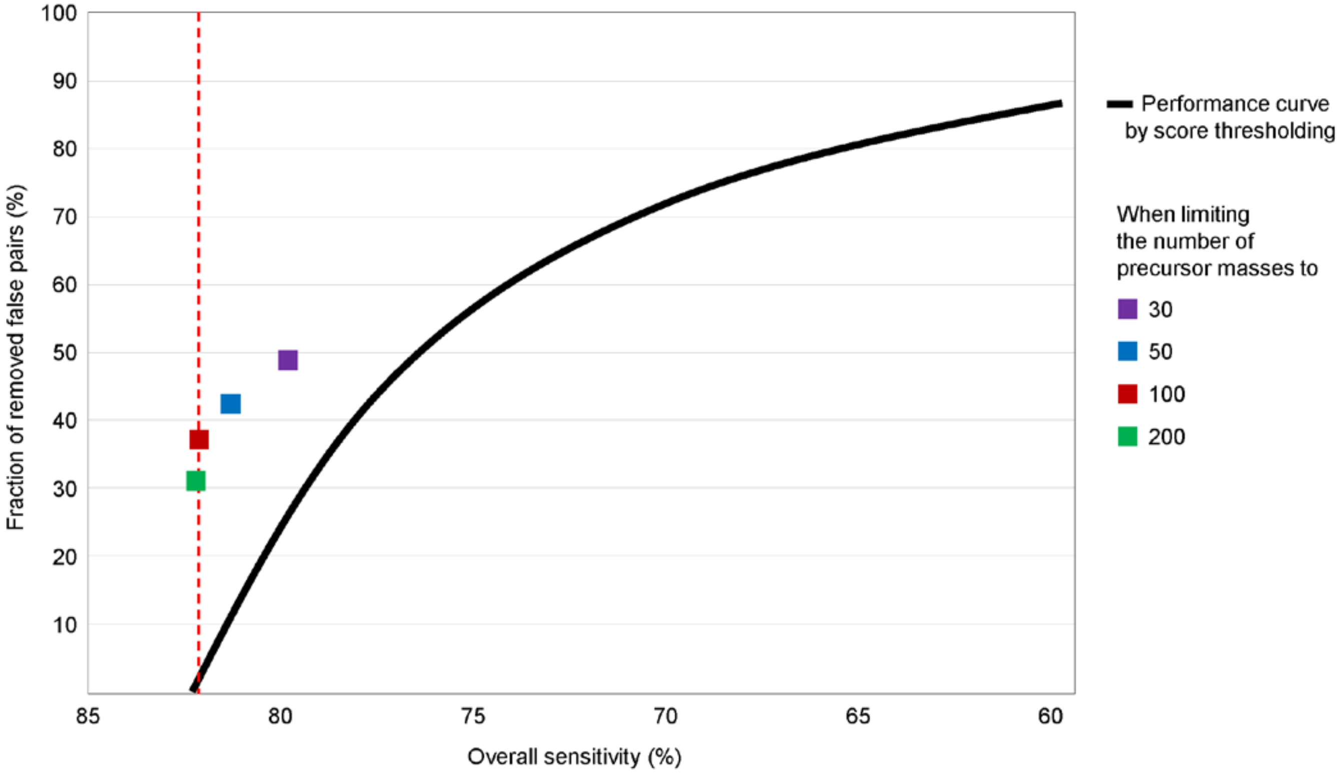

Removing false spectral pairs to split mixed subnetworks. When constructing spectral networks from spectral pairs detected using Align-GF *p*-values for spectrum-spectrum alignments, we found that the accuracy of the resulting subnetworks could be substantially improved by limiting the number of unique precursor ion masses in each subnetwork. Restricting this number to a maximum of 100, we found that 37% of false variant pairs were removed from the original network while removing only 0.2% of true variant pairs (shown as a red rectangle). In contrast, the black curve shows the fraction of removed false variant pairs according to sensitivity by *p*-value thresholding, which would eliminate only ~2% of false pairs with the same sensitivity loss (at dotted red line). In spectral networks, removing false pairs may be more important than retaining more true pairs because removing a single false pair could prevent the propagated misannotation of many spectra, while a lost true pair may be compensated because two spectra in the pair can still remain connected through a different path in the subnetwork by using other spectra from the same peptide family.

Figure S3.

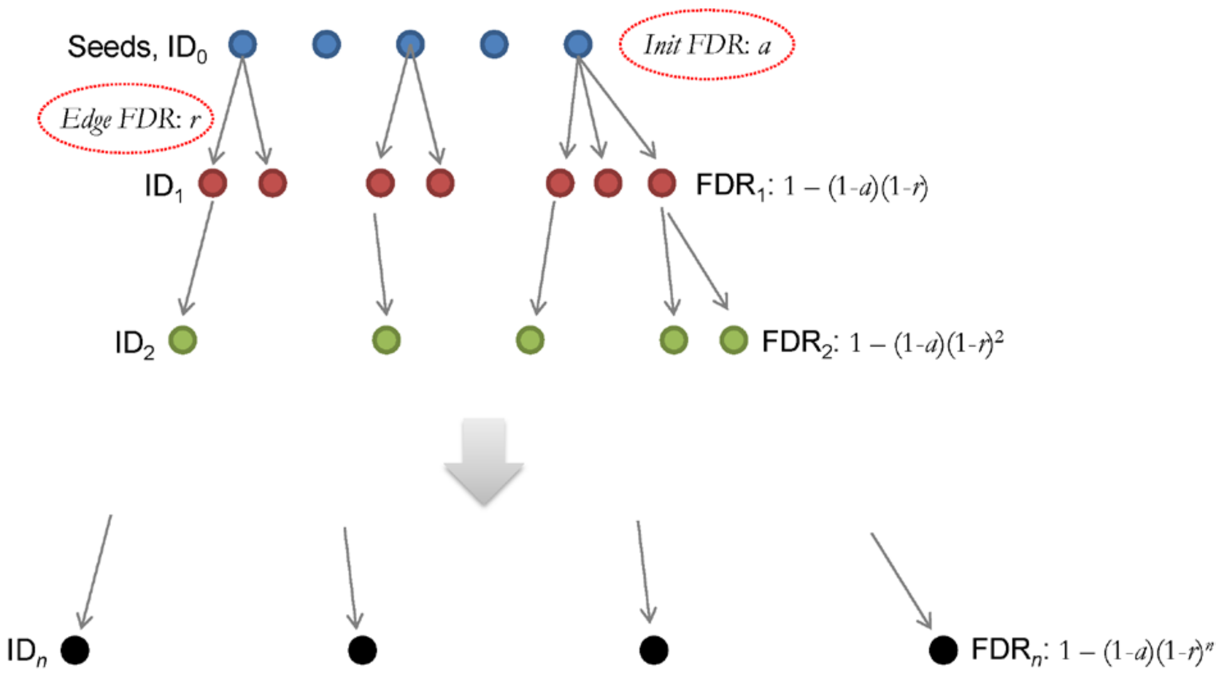

$$aggregateFDR = \frac{\sum_{k=0}^{n} ID_k \times (1-(1-a)(1-r)^k)}{\sum_{k=0}^{n} ID_k}$$

ID_k = is the number of identifications added at *k*-th propagation

FDR estimation for propagation in a spectral network. *Init* FDR *a* is the FDR in seed identification and is determined when the seeds are obtained; *Edge* FDR *r* is the FDR in spectral pairs and is calculated using spectral pairs annotated by the seeds. The two FDRs are involved in the overall error of propagation. After the first propagation, FDR_1 is calculated as the probability that its seed or the propagation edge is incorrect. Added propagations also introduce edge errors to the FDR calculation, reflecting that highly-modified peptides are more likely to include more false-positives. FDR of the overall identifications is estimated by adding up the errors from all propagation steps.

Figure S4.

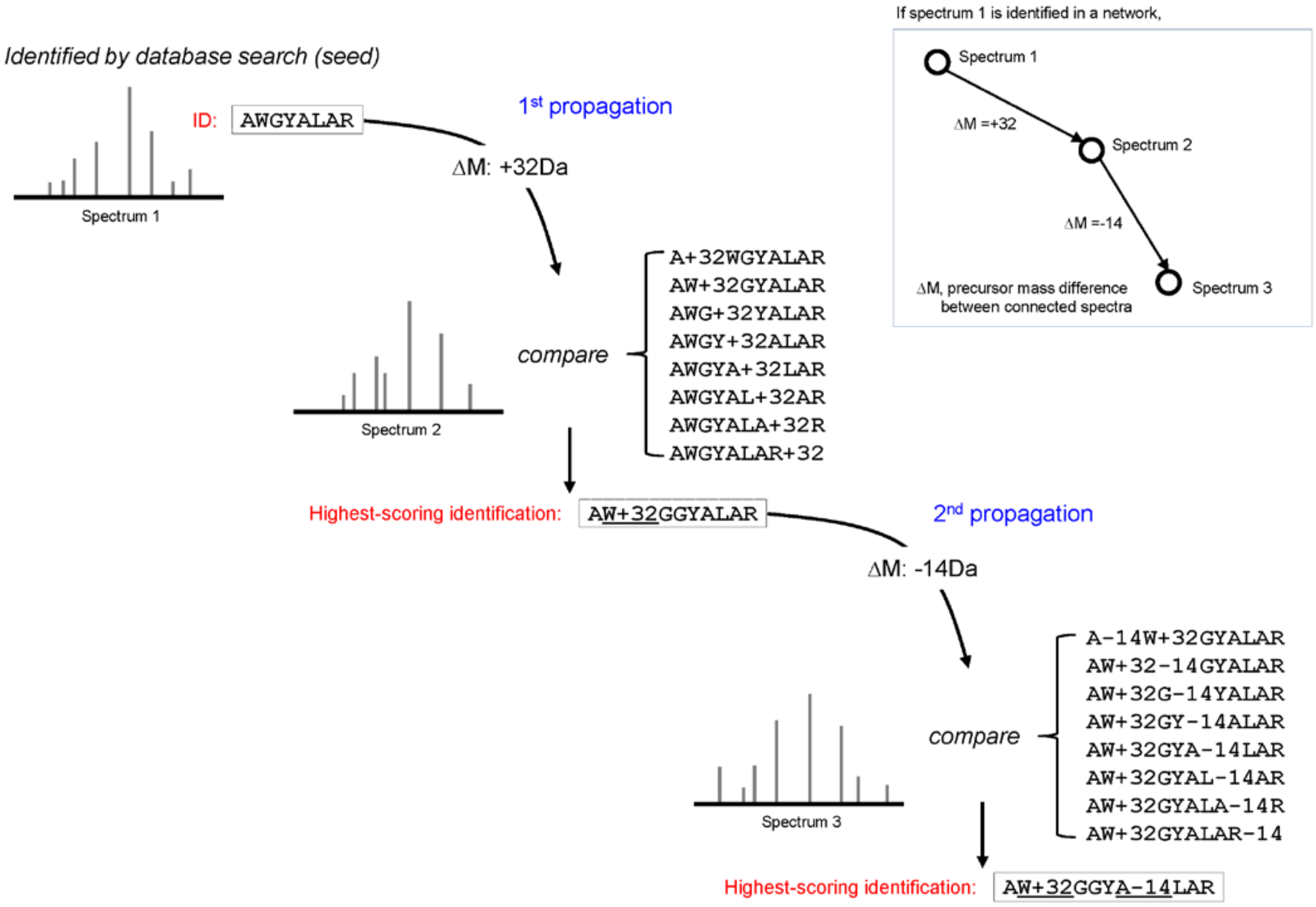

Localization of modifications. In a network consisting of three spectra, if spectrum 1 is identified as 'AWGGYALAR', the peptide sequence is propagated to spectrum 2 with the mass difference of +32 Da. Our algorithm considers the modification mass on all amino acids of the propagated sequence, compares all theoretical spectra to spectrum 2, and selects the highest-scoring identification, 'AW+32GGYALAR'. Similarly, in propagation from spectrum 2 to 3, the modification mass of – 14 Da is considered on all possible locations of the identification of spectrum 2, ' AW+32GGYALAR', as shown in this figure.

Figure S5.

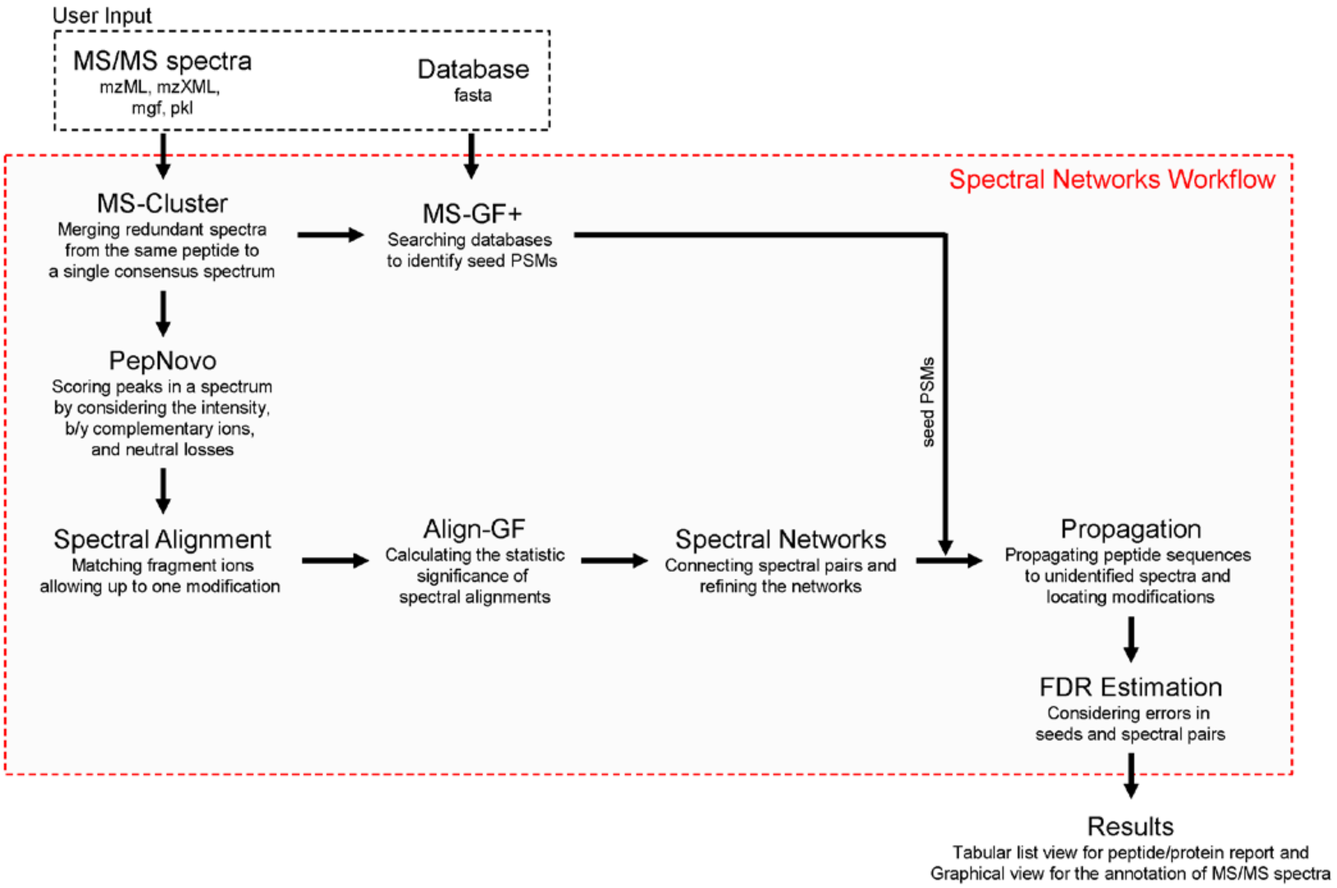

Spectral networks workflow. Spectral networks workflow integrates software and algorithms into a web-based pipeline, which is available at http://proteomics.ucsd.edu/software.

Figure S6.

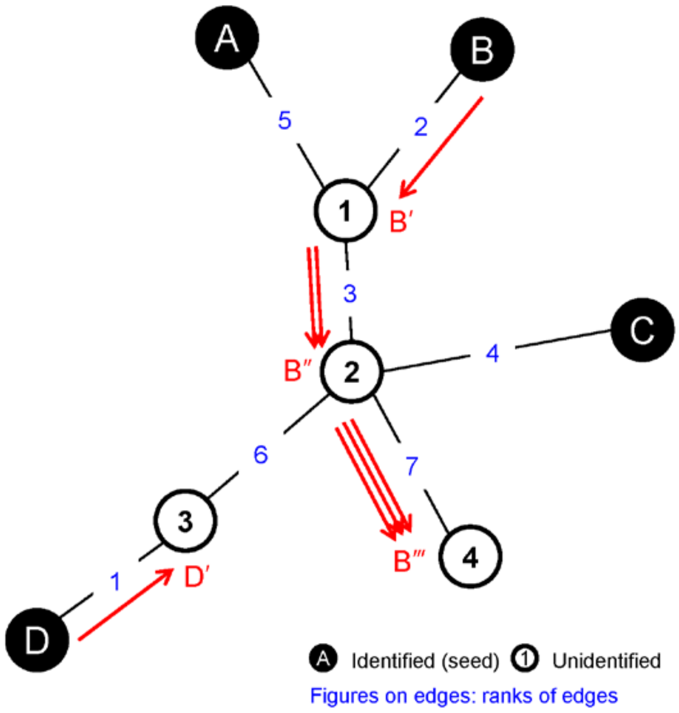

Propagation procedure. This figure illustrates the propagation procedure on a simple network. The black nodes represent initial seeds (annotated spectra), and the white nodes represent non-annotated spectra. Numbers on edges are ranks of edges based on Align-GF *p*-value; the rank of the best edge is 1. The propagation rule is that a non-annotated node is propagated through the best incoming edge. We use an iterative procedure where at each step the best edge between an annotated node and an unannotated node is used to propagate the annotation. For example, at the beginning, node 3 is annotated as D' from seed D, the next step annotates node 1 with B', and this process is iterated until no more nodes can be annotated or the FDR threshold is crossed. Red arrows correspond to the propagation, and repeated arrows represent cumulative propagations.

Figure S7.

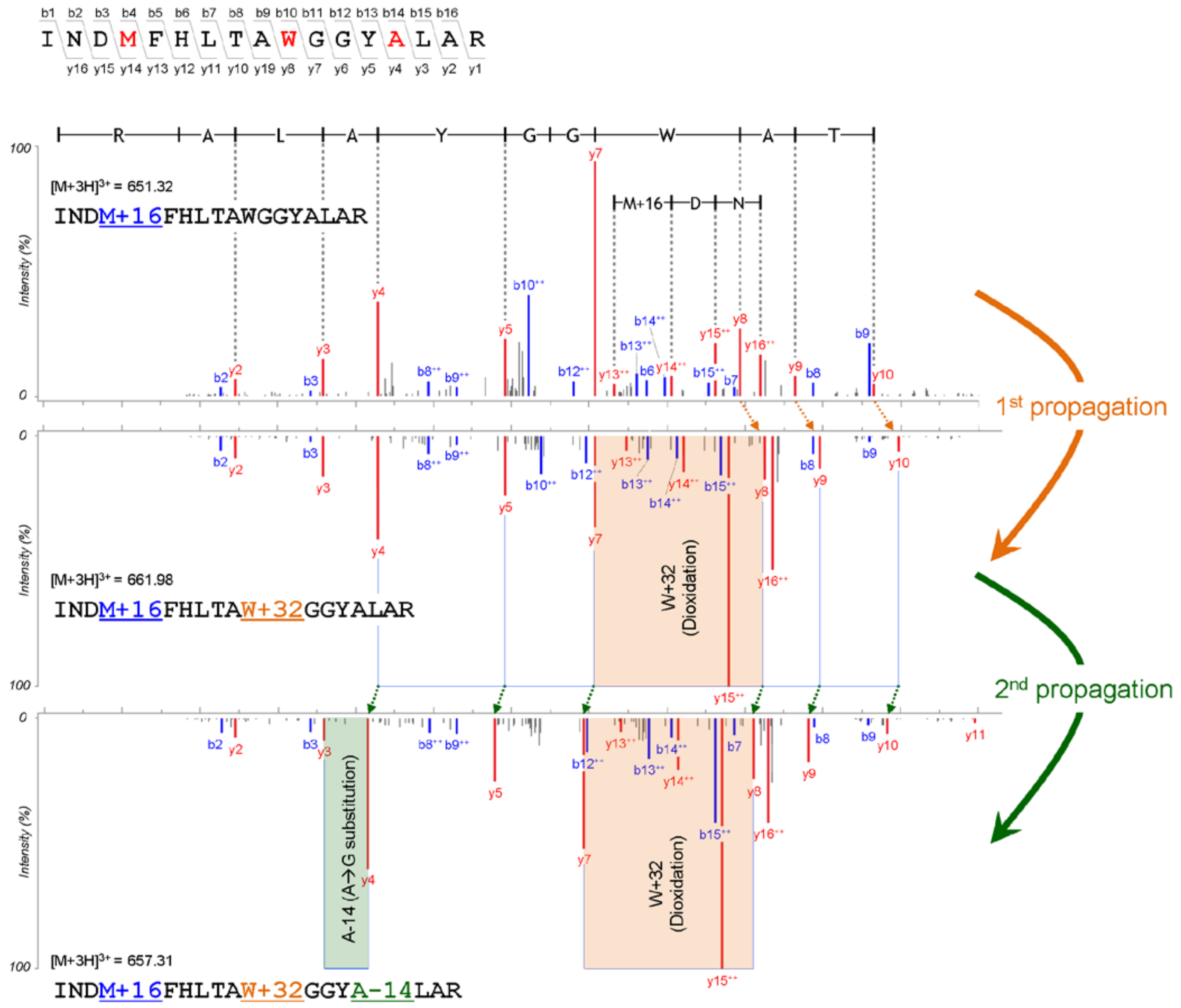

Identification of multiply-modified peptides by iterative propagation. Annotated MS/MS spectra of singly-modified (top), doubly-modified (middle), and triply-modified (bottom) forms of a peptide are shown. Starting with the seed, the first propagation identified di-oxidized Trp and then the second propagation identified Gly substituted for Ala in order. The search space of spectral networks for identifying multiply-modified peptides is much smaller than that of other approaches to directly identify multiply-modified peptides using the whole database sequence. In addition, the identified multiply-modified peptides are further supported by multiple spectra from different modification states of a peptide in the same spectral network, thus increasing the sensitivity and reliability of spectral networks identifications.

Figure S8.

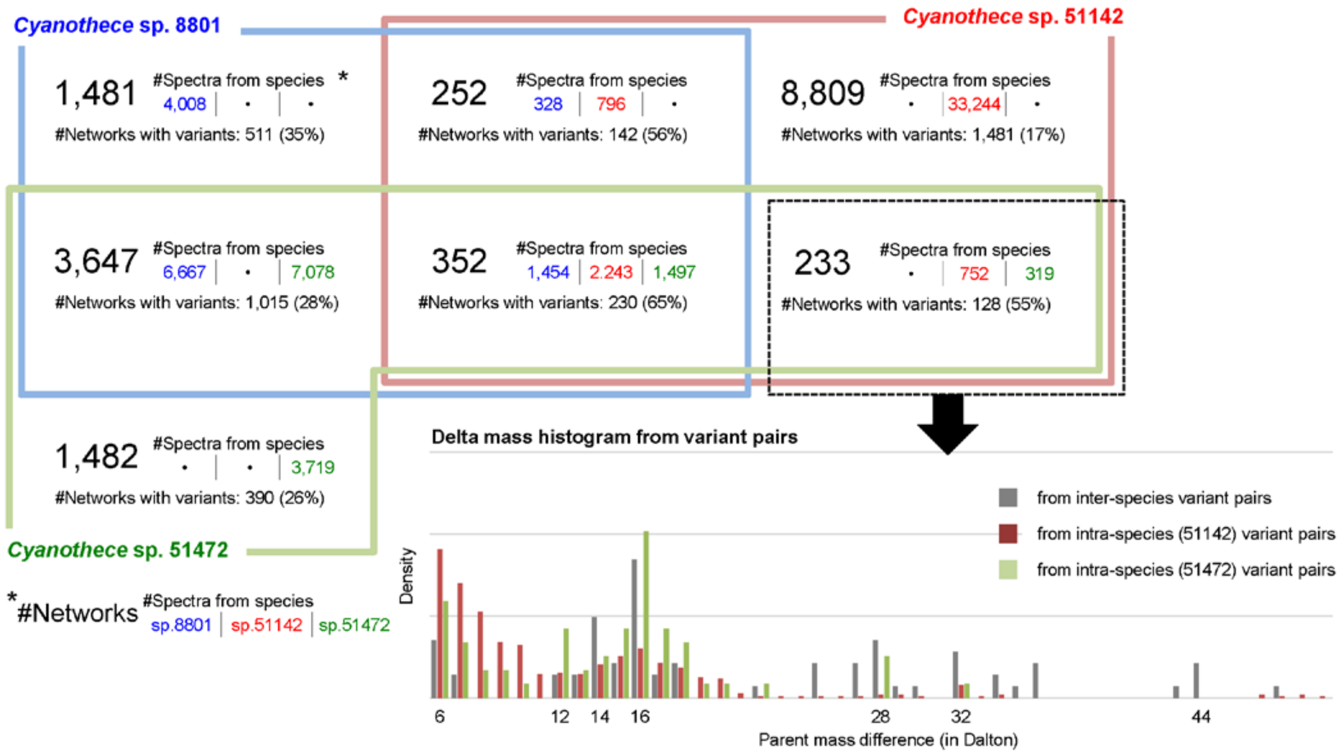

Unidentified spectral networks. This Venn diagram classifies 16,256 unidentified subnetworks into 7 groups by combinations of species based upon the origin of the spectra in each subnetwork. For each group, two properties are shown: 1) *#Spectra from species* is the number of spectra in the group from *Cyanothece* sp. 8801 at the first column, 51142 at the second, and 51472 at the third, respectively; 2) *#Networks with variants* is the number of subnetworks that include variant pairs – spectral pairs in which the parent mass difference between the two spectra is larger than 5 Da. In the right bottom, the delta mass histogram is shown for variant pairs in the group that includes spectra from species 51142 and 51472 (dotted rectangle), where the gray distribution was calculated from inter-species pairs. These unidentified subnetworks contain no identified spectra, but suggest the presence of novel peptides supported by at least two different overlapping peptide variants, in particular for subnetworks consisting of spectra from multiple species.
